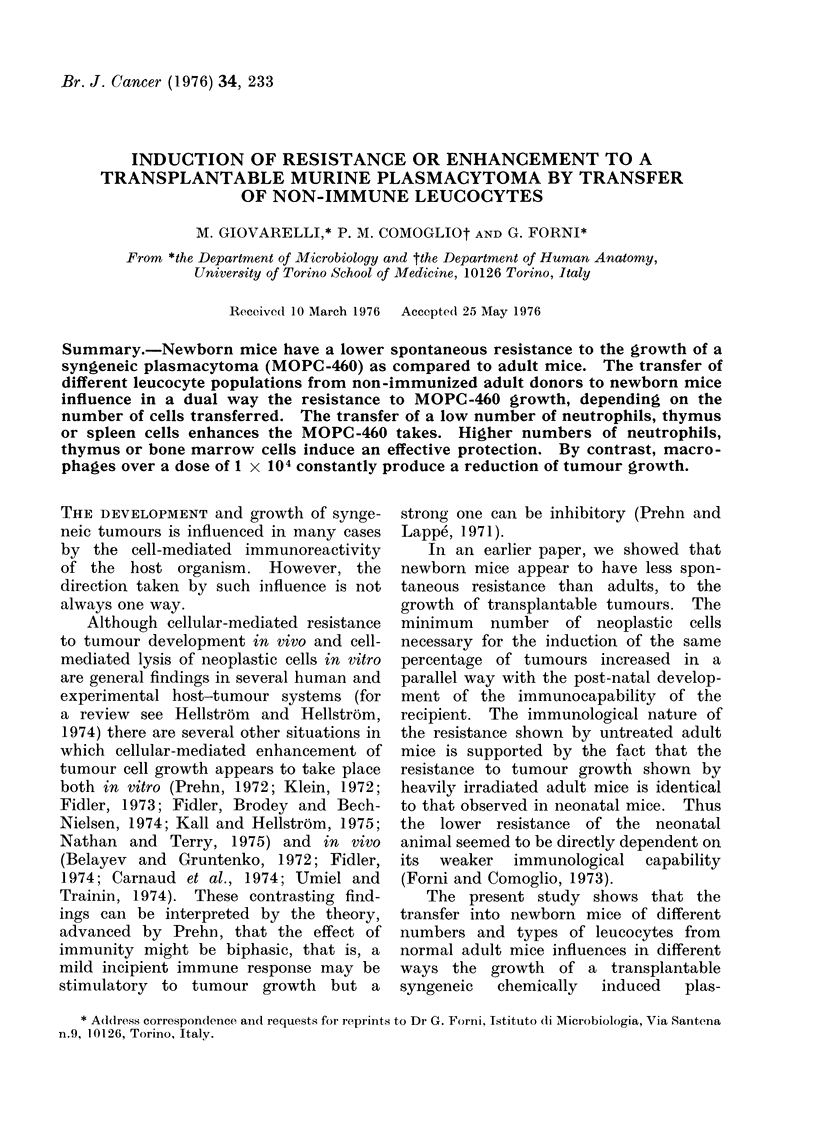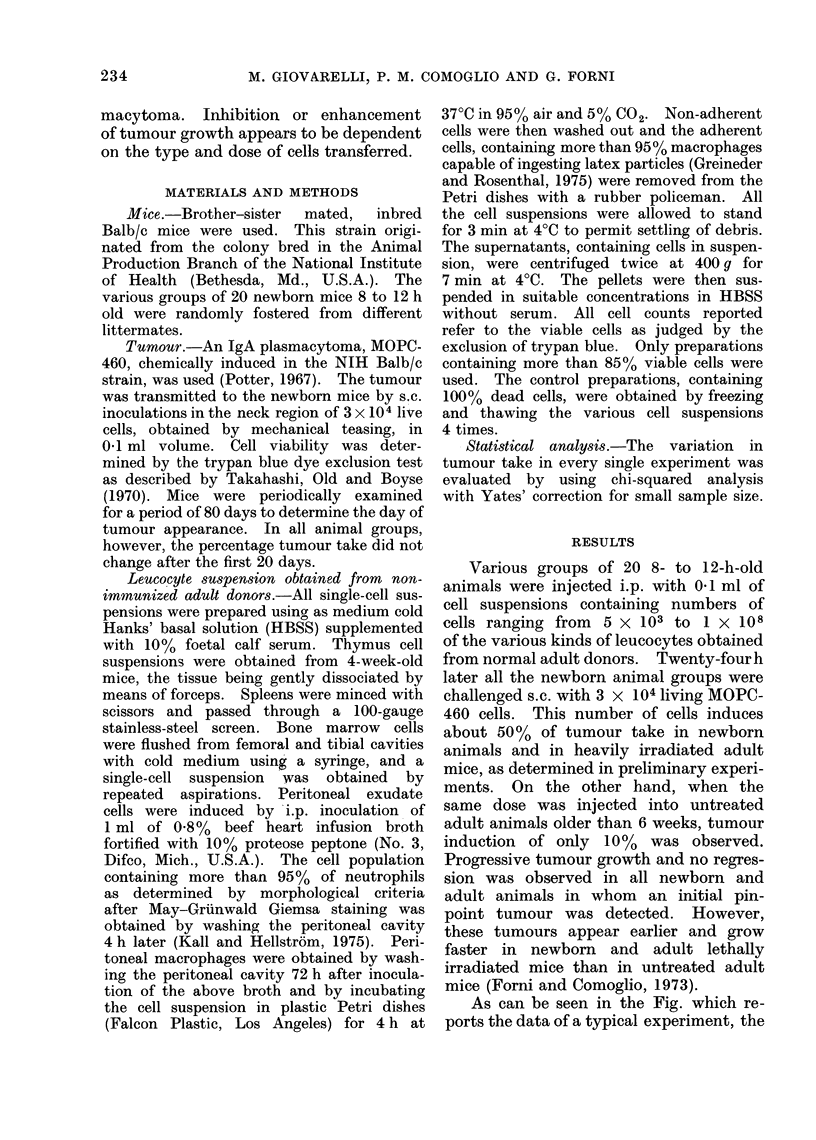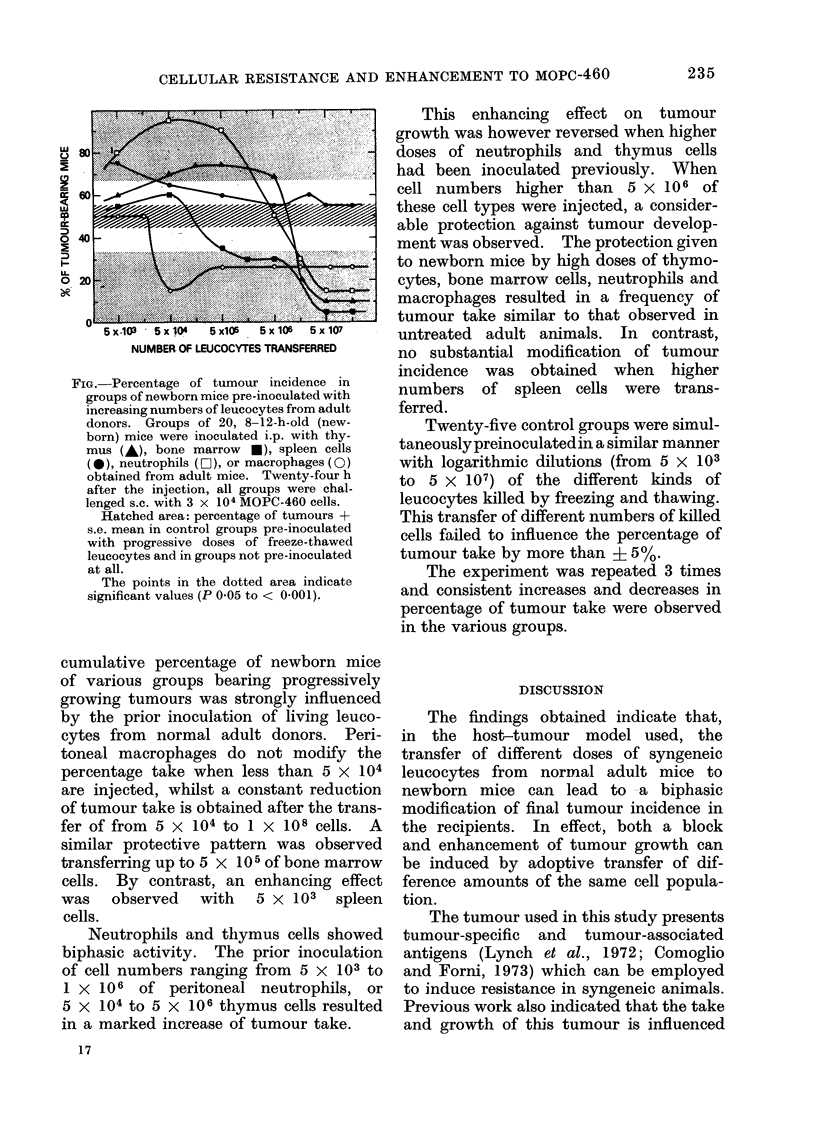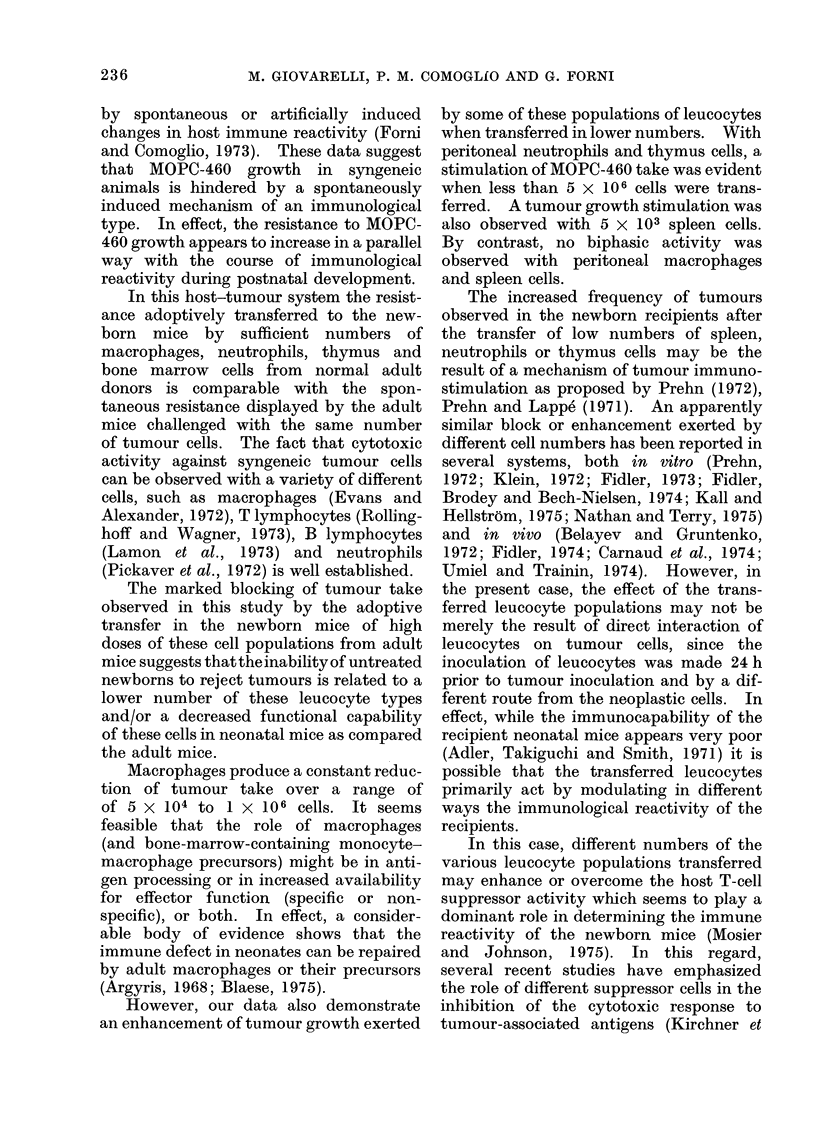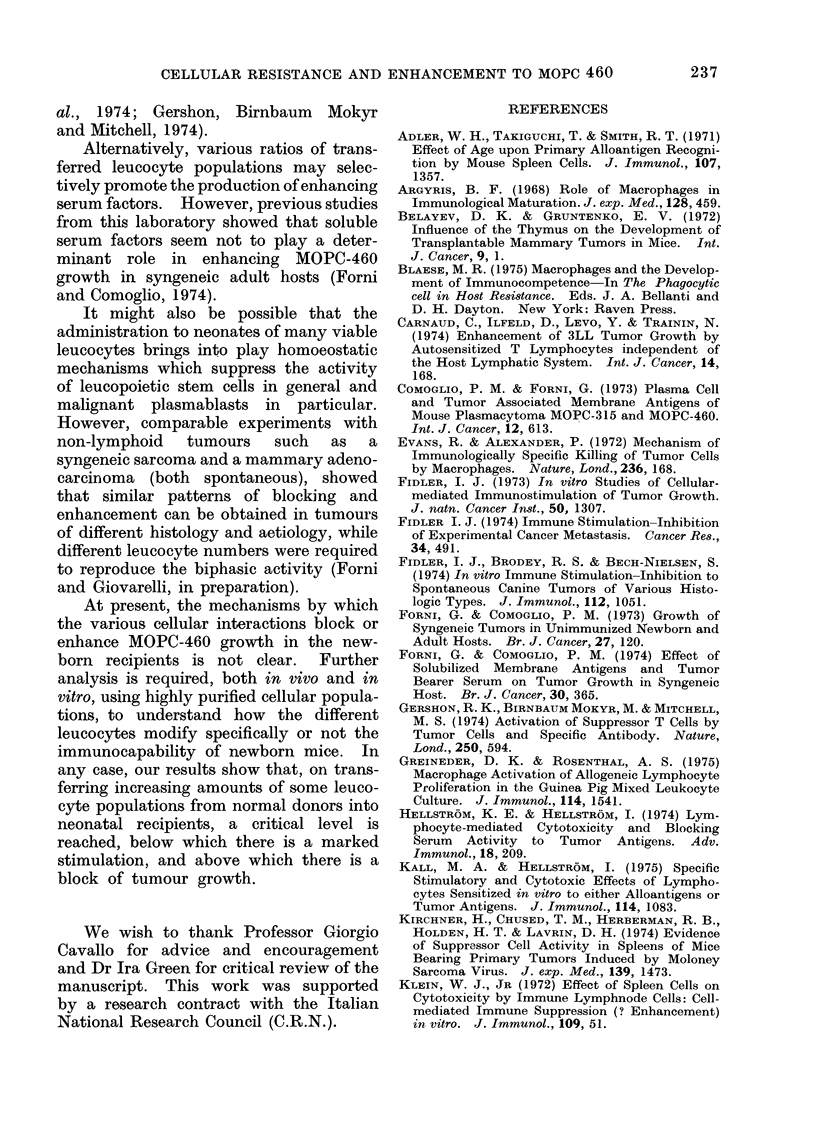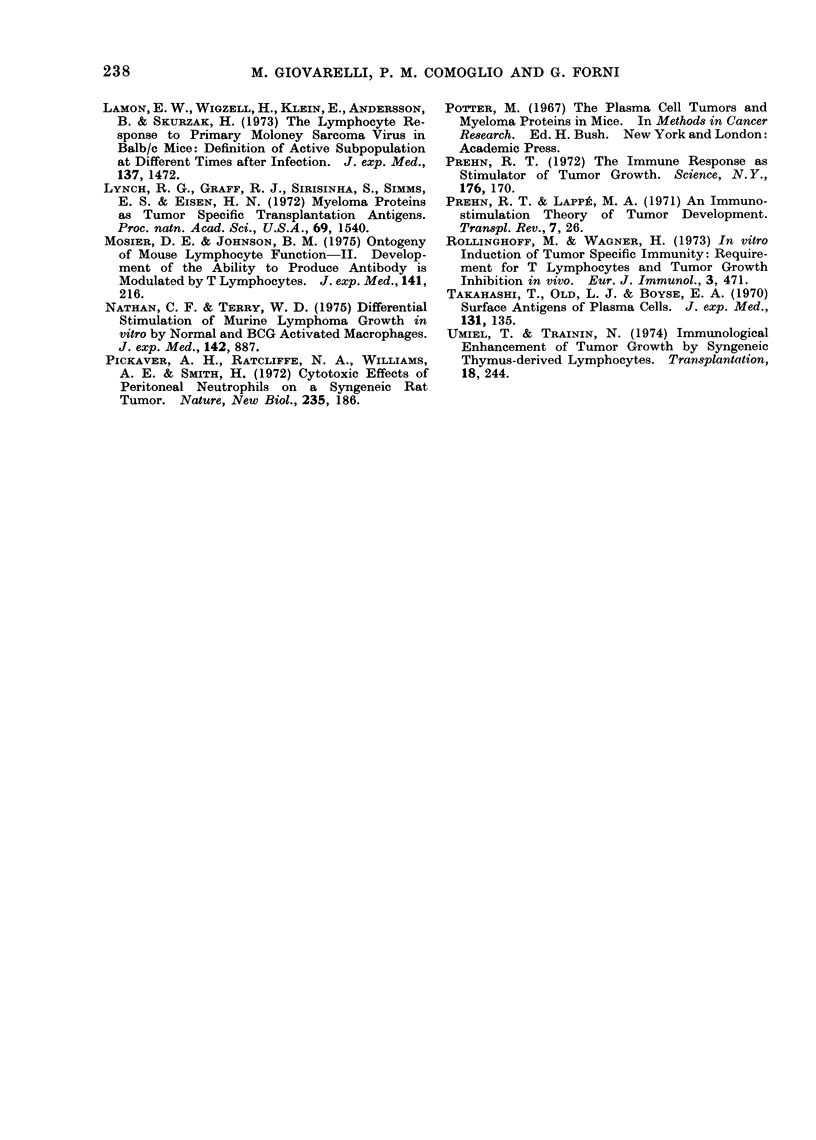# Induction of resistance or enhancement to a transplantable murine plasmacytoma by transfer of non-immune leucocytes.

**DOI:** 10.1038/bjc.1976.157

**Published:** 1976-09

**Authors:** M. Giovarelli, P. M. Comoglio, G. Forni

## Abstract

Newborn mice have a lower spontaneous resistance to the growth of a syngeneic plasmacytoma (MOPC-460) as compared to adult mice. The transfer of different leucocyte populations from non-immunized adult donors to newborn mice influence in a dual way the resistance to MOPC-460 growth, depending on the number of cells transferred. The transfer of a low number of neutrophils, thymus or spleen cells enhances the MOPC-460 takes. Higher numbers of neutrophils, thymus or bone marrow cells induce an effective protenction. By contrast, macrophages over a dose of 1 X 10(4) constantly produce a reduction of tumour growth.


*Br. J. Cancer* (1976) **34**, 233

### INDUCTION OF RESISTANCE OR ENHANCEMENT TO A TRANSPLANTABLE MURINE PLASMACYTOMA BY TRANSFER OF NON-IMMUNE LEUCOCYTES

M. GIOVARELLI,* P. M. COMOGLIO† AND G. FORNI*

*From *the Department of Microbiology and †the Department of Human Anatomy, University of Torino School of Medicine,* 10126 *Torino, Italy*

Received 10 March 1976 Accepted 25 May 1976

**Summary.—Newborn mice have a lower spontaneous resistance to the growth of a syngeneic plasmacytoma (MOPC-460) as compared to adult mice. The transfer of different leucocyte populations from non-immunized adult donors to newborn mice influence in a dual way the resistance to MOPC-460 growth, depending on the number of cells transferred. The transfer of a low number of neutrophils, thymus or spleen cells enhances the MOPC-460 takes. Higher numbers of neutrophils, thymus or bone marrow cells induce an effective protection. By contrast, macrophages over a dose of $1 \times 10^4$ constantly produce a reduction of tumour growth.**

THE DEVELOPMENT and growth of syngeneic tumours is influenced in many cases by the cell-mediated immunoreactivity of the host organism. However, the direction taken by such influence is not always one way.

Although cellular-mediated resistance to tumour development *in vivo* and cell-mediated lysis of neoplastic cells *in vitro* are general findings in several human and experimental host–tumour systems (for a review see Hellström and Hellström, 1974) there are several other situations in which cellular-mediated enhancement of tumour cell growth appears to take place both *in vitro* (Prehn, 1972; Klein, 1972; Fidler, 1973; Fidler, Brodey and Bech-Nielsen, 1974; Kall and Hellström, 1975; Nathan and Terry, 1975) and *in vivo* (Belayev and Gruntenko, 1972; Fidler, 1974; Carnaud *et al.*, 1974; Umiel and Trainin, 1974). These contrasting findings can be interpreted by the theory, advanced by Prehn, that the effect of immunity might be biphasic, that is, a mild incipient immune response may be stimulatory to tumour growth but a strong one can be inhibitory (Prehn and Lappé, 1971).

In an earlier paper, we showed that newborn mice appear to have less spontaneous resistance than adults, to the growth of transplantable tumours. The minimum number of neoplastic cells necessary for the induction of the same percentage of tumours increased in a parallel way with the post-natal development of the immunocapability of the recipient. The immunological nature of the resistance shown by untreated adult mice is supported by the fact that the resistance to tumour growth shown by heavily irradiated adult mice is identical to that observed in neonatal mice. Thus the lower resistance of the neonatal animal seemed to be directly dependent on its weaker immunological capability (Forni and Comoglio, 1973).

The present study shows that the transfer into newborn mice of different numbers and types of leucocytes from normal adult mice influences in different ways the growth of a transplantable syngeneic chemically induced plas-

* Address correspondence and requests for reprints to Dr G. Forni, Istituto di Microbiologia, Via Santena n.9, 10126, Torino, Italy.

macytoma. Inhibition or enhancement of tumour growth appears to be dependent on the type and dose of cells transferred.

### MATERIALS AND METHODS

*Mice*.—Brother–sister mated, inbred Balb/c mice were used. This strain originated from the colony bred in the Animal Production Branch of the National Institute of Health (Bethesda, Md., U.S.A.). The various groups of 20 newborn mice 8 to 12 h old were randomly fostered from different littermates.

*Tumour*.—An IgA plasmacytoma, MOPC-460, chemically induced in the NIH Balb/c strain, was used (Potter, 1967). The tumour was transmitted to the newborn mice by s.c. inoculations in the neck region of $3 \times 10^4$ live cells, obtained by mechanical teasing, in 0·1 ml volume. Cell viability was determined by the trypan blue dye exclusion test as described by Takahashi, Old and Boyse (1970). Mice were periodically examined for a period of 80 days to determine the day of tumour appearance. In all animal groups, however, the percentage tumour take did not change after the first 20 days.

*Leucocyte suspension obtained from non-immunized adult donors*.—All single-cell suspensions were prepared using as medium cold Hanks' basal solution (HBSS) supplemented with 10% foetal calf serum. Thymus cell suspensions were obtained from 4-week-old mice, the tissue being gently dissociated by means of forceps. Spleens were minced with scissors and passed through a 100-gauge stainless-steel screen. Bone marrow cells were flushed from femoral and tibial cavities with cold medium using a syringe, and a single-cell suspension was obtained by repeated aspirations. Peritoneal exudate cells were induced by i.p. inoculation of 1 ml of 0·8% beef heart infusion broth fortified with 10% proteose peptone (No. 3, Difco, Mich., U.S.A.). The cell population containing more than 95% of neutrophils as determined by morphological criteria after May–Grünwald Giemsa staining was obtained by washing the peritoneal cavity 4 h later (Kall and Hellström, 1975). Peritoneal macrophages were obtained by washing the peritoneal cavity 72 h after inoculation of the above broth and by incubating the cell suspension in plastic Petri dishes (Falcon Plastic, Los Angeles) for 4 h at 37°C in 95% air and 5% $CO_2$. Non-adherent cells were then washed out and the adherent cells, containing more than 95% macrophages capable of ingesting latex particles (Greineder and Rosenthal, 1975) were removed from the Petri dishes with a rubber policeman. All the cell suspensions were allowed to stand for 3 min at 4°C to permit settling of debris. The supernatants, containing cells in suspension, were centrifuged twice at 400 *g* for 7 min at 4°C. The pellets were then suspended in suitable concentrations in HBSS without serum. All cell counts reported refer to the viable cells as judged by the exclusion of trypan blue. Only preparations containing more than 85% viable cells were used. The control preparations, containing 100% dead cells, were obtained by freezing and thawing the various cell suspensions 4 times.

*Statistical analysis*.—The variation in tumour take in every single experiment was evaluated by using chi-squared analysis with Yates' correction for small sample size.

### RESULTS

Various groups of 20 8- to 12-h-old animals were injected i.p. with 0·1 ml of cell suspensions containing numbers of cells ranging from $5 \times 10^3$ to $1 \times 10^8$ of the various kinds of leucocytes obtained from normal adult donors. Twenty-four h later all the newborn animal groups were challenged s.c. with $3 \times 10^4$ living MOPC-460 cells. This number of cells induces about 50% of tumour take in newborn animals and in heavily irradiated adult mice, as determined in preliminary experiments. On the other hand, when the same dose was injected into untreated adult animals older than 6 weeks, tumour induction of only 10% was observed. Progressive tumour growth and no regression was observed in all newborn and adult animals in whom an initial pin-point tumour was detected. However, these tumours appear earlier and grow faster in newborn and adult lethally irradiated mice than in untreated adult mice (Forni and Comoglio, 1973).

As can be seen in the Fig. which reports the data of a typical experiment, the

FIG.—Percentage of tumour incidence in groups of newborn mice pre-inoculated with increasing numbers of leucocytes from adult donors. Groups of 20, 8–12-h-old (newborn) mice were inoculated i.p. with thymus (▲), bone marrow ■), spleen cells (●), neutrophils (□), or macrophages (○) obtained from adult mice. Twenty-four h after the injection, all groups were challenged s.c. with $3 \times 10^4$ MOPC-460 cells.

Hatched area: percentage of tumours + s.e. mean in control groups pre-inoculated with progressive doses of freeze-thawed leucocytes and in groups not pre-inoculated at all.

The points in the dotted area indicate significant values ($P$ 0·05 to $< 0{\cdot}001$).

cumulative percentage of newborn mice of various groups bearing progressively growing tumours was strongly influenced by the prior inoculation of living leucocytes from normal adult donors. Peritoneal macrophages do not modify the percentage take when less than $5 \times 10^4$ are injected, whilst a constant reduction of tumour take is obtained after the transfer of from $5 \times 10^4$ to $1 \times 10^8$ cells. A similar protective pattern was observed transferring up to $5 \times 10^5$ of bone marrow cells. By contrast, an enhancing effect was observed with $5 \times 10^3$ spleen cells.

Neutrophils and thymus cells showed biphasic activity. The prior inoculation of cell numbers ranging from $5 \times 10^3$ to $1 \times 10^6$ of peritoneal neutrophils, or $5 \times 10^4$ to $5 \times 10^6$ thymus cells resulted in a marked increase of tumour take.

This enhancing effect on tumour growth was however reversed when higher doses of neutrophils and thymus cells had been inoculated previously. When cell numbers higher than $5 \times 10^6$ of these cell types were injected, a considerable protection against tumour development was observed. The protection given to newborn mice by high doses of thymocytes, bone marrow cells, neutrophils and macrophages resulted in a frequency of tumour take similar to that observed in untreated adult animals. In contrast, no substantial modification of tumour incidence was obtained when higher numbers of spleen cells were transferred.

Twenty-five control groups were simultaneously preinoculated in a similar manner with logarithmic dilutions (from $5 \times 10^3$ to $5 \times 10^7$) of the different kinds of leucocytes killed by freezing and thawing. This transfer of different numbers of killed cells failed to influence the percentage of tumour take by more than $\pm 5\%$.

The experiment was repeated 3 times and consistent increases and decreases in percentage of tumour take were observed in the various groups.

### DISCUSSION

The findings obtained indicate that, in the host–tumour model used, the transfer of different doses of syngeneic leucocytes from normal adult mice to newborn mice can lead to a biphasic modification of final tumour incidence in the recipients. In effect, both a block and enhancement of tumour growth can be induced by adoptive transfer of difference amounts of the same cell population.

The tumour used in this study presents tumour-specific and tumour-associated antigens (Lynch *et al.*, 1972; Comoglio and Forni, 1973) which can be employed to induce resistance in syngeneic animals. Previous work also indicated that the take and growth of this tumour is influenced by spontaneous or artificially induced changes in host immune reactivity (Forni and Comoglio, 1973). These data suggest that MOPC-460 growth in syngeneic animals is hindered by a spontaneously induced mechanism of an immunological type. In effect, the resistance to MOPC-460 growth appears to increase in a parallel way with the course of immunological reactivity during postnatal development.

In this host–tumour system the resistance adoptively transferred to the newborn mice by sufficient numbers of macrophages, neutrophils, thymus and bone marrow cells from normal adult donors is comparable with the spontaneous resistance displayed by the adult mice challenged with the same number of tumour cells. The fact that cytotoxic activity against syngeneic tumour cells can be observed with a variety of different cells, such as macrophages (Evans and Alexander, 1972), T lymphocytes (Rollinghoff and Wagner, 1973), B lymphocytes (Lamon *et al.*, 1973) and neutrophils (Pickaver *et al.*, 1972) is well established.

The marked blocking of tumour take observed in this study by the adoptive transfer in the newborn mice of high doses of these cell populations from adult mice suggests that the inability of untreated newborns to reject tumours is related to a lower number of these leucocyte types and/or a decreased functional capability of these cells in neonatal mice as compared the adult mice.

Macrophages produce a constant reduction of tumour take over a range of of $5 \times 10^4$ to $1 \times 10^6$ cells. It seems feasible that the role of macrophages (and bone-marrow-containing monocyte–macrophage precursors) might be in antigen processing or in increased availability for effector function (specific or nonspecific), or both. In effect, a considerable body of evidence shows that the immune defect in neonates can be repaired by adult macrophages or their precursors (Argyris, 1968; Blaese, 1975).

However, our data also demonstrate an enhancement of tumour growth exerted by some of these populations of leucocytes when transferred in lower numbers. With peritoneal neutrophils and thymus cells, a stimulation of MOPC-460 take was evident when less than $5 \times 10^6$ cells were transferred. A tumour growth stimulation was also observed with $5 \times 10^3$ spleen cells. By contrast, no biphasic activity was observed with peritoneal macrophages and spleen cells.

The increased frequency of tumours observed in the newborn recipients after the transfer of low numbers of spleen, neutrophils or thymus cells may be the result of a mechanism of tumour immunostimulation as proposed by Prehn (1972), Prehn and Lappé (1971). An apparently similar block or enhancement exerted by different cell numbers has been reported in several systems, both *in vitro* (Prehn, 1972; Klein, 1972; Fidler, 1973; Fidler, Brodey and Bech-Nielsen, 1974; Kall and Hellström, 1975; Nathan and Terry, 1975) and *in vivo* (Belayev and Gruntenko, 1972; Fidler, 1974; Carnaud *et al.*, 1974; Umiel and Trainin, 1974). However, in the present case, the effect of the transferred leucocyte populations may not be merely the result of direct interaction of leucocytes on tumour cells, since the inoculation of leucocytes was made 24 h prior to tumour inoculation and by a different route from the neoplastic cells. In effect, while the immunocapability of the recipient neonatal mice appears very poor (Adler, Takiguchi and Smith, 1971) it is possible that the transferred leucocytes primarily act by modulating in different ways the immunological reactivity of the recipients.

In this case, different numbers of the various leucocyte populations transferred may enhance or overcome the host T-cell suppressor activity which seems to play a dominant role in determining the immune reactivity of the newborn mice (Mosier and Johnson, 1975). In this regard, several recent studies have emphasized the role of different suppressor cells in the inhibition of the cytotoxic response to tumour-associated antigens (Kirchner *et*

*al.*, 1974; Gershon, Birnbaum Mokyr and Mitchell, 1974).

Alternatively, various ratios of transferred leucocyte populations may selectively promote the production of enhancing serum factors. However, previous studies from this laboratory showed that soluble serum factors seem not to play a determinant role in enhancing MOPC-460 growth in syngeneic adult hosts (Forni and Comoglio, 1974).

It might also be possible that the administration to neonates of many viable leucocytes brings into play homoeostatic mechanisms which suppress the activity of leucopoietic stem cells in general and malignant plasmablasts in particular. However, comparable experiments with non-lymphoid tumours such as a syngeneic sarcoma and a mammary adenocarcinoma (both spontaneous), showed that similar patterns of blocking and enhancement can be obtained in tumours of different histology and aetiology, while different leucocyte numbers were required to reproduce the biphasic activity (Forni and Giovarelli, in preparation).

At present, the mechanisms by which the various cellular interactions block or enhance MOPC-460 growth in the newborn recipients is not clear. Further analysis is required, both *in vivo* and *in vitro*, using highly purified cellular populations, to understand how the different leucocytes modify specifically or not the immunocapability of newborn mice. In any case, our results show that, on transferring increasing amounts of some leucocyte populations from normal donors into neonatal recipients, a critical level is reached, below which there is a marked stimulation, and above which there is a block of tumour growth.

We wish to thank Professor Giorgio Cavallo for advice and encouragement and Dr Ira Green for critical review of the manuscript. This work was supported by a research contract with the Italian National Research Council (C.R.N.).

### REFERENCES

Adler, W. H., Takiguchi, T. & Smith, R. T. (1971) Effect of Age upon Primary Alloantigen Recognition by Mouse Spleen Cells. *J. Immunol.*, **107**, 1357.

Argyris, B. F. (1968) Role of Macrophages in Immunological Maturation. *J. exp. Med.*, **128**, 459.

Belayev, D. K. & Gruntenko, E. V. (1972) Influence of the Thymus on the Development of Transplantable Mammary Tumors in Mice. *Int. J. Cancer*, **9**, 1.

Blaese, M. R. (1975) Macrophages and the Development of Immunocompetence—In *The Phagocytic cell in Host Resistance*. Eds. J. A. Bellanti and D. H. Dayton. New York: Raven Press.

Carnaud, C., Ilfeld, D., Levo, Y. & Trainin, N. (1974) Enhancement of 3LL Tumor Growth by Autosensitized T Lymphocytes independent of the Host Lymphatic System. *Int. J. Cancer*, **14**, 168.

Comoglio, P. M. & Forni, G. (1973) Plasma Cell and Tumor Associated Membrane Antigens of Mouse Plasmacytoma MOPC-315 and MOPC-460. *Int. J. Cancer*, **12**, 613.

Evans, R. & Alexander, P. (1972) Mechanism of Immunologically Specific Killing of Tumor Cells by Macrophages. *Nature, Lond.*, **236**, 168.

Fidler, I. J. (1973) *In vitro* Studies of Cellular-mediated Immunostimulation of Tumor Growth. *J. natn. Cancer Inst.*, **50**, 1307.

Fidler I. J. (1974) Immune Stimulation–Inhibition of Experimental Cancer Metastasis. *Cancer Res.*, **34**, 491.

Fidler, I. J., Brodey, R. S. & Bech-Nielsen, S. (1974) *In vitro* Immune Stimulation–Inhibition to Spontaneous Canine Tumors of Various Histologic Types. *J. Immunol.*, **112**, 1051.

Forni, G. & Comoglio, P. M. (1973) Growth of Syngeneic Tumors in Unimmunized Newborn and Adult Hosts. *Br. J. Cancer*, **27**, 120.

Forni, G. & Comoglio, P. M. (1974) Effect of Solubilized Membrane Antigens and Tumor Bearer Serum on Tumor Growth in Syngeneic Host. *Br. J. Cancer*, **30**, 365.

Gershon, R. K., Birnbaum Mokyr, M. & Mitchell, M. S. (1974) Activation of Suppressor T Cells by Tumor Cells and Specific Antibody. *Nature, Lond.*, **250**, 594.

Greineder, D. K. & Rosenthal, A. S. (1975) Macrophage Activation of Allogeneic Lymphocyte Proliferation in the Guinea Pig Mixed Leukocyte Culture. *J. Immunol.*, **114**, 1541.

Hellström, K. E. & Hellström, I. (1974) Lymphocyte-mediated Cytotoxicity and Blocking Serum Activity to Tumor Antigens. *Adv. Immunol.*, **18**, 209.

Kall, M. A. & Hellström, I. (1975) Specific Stimulatory and Cytotoxic Effects of Lymphocytes Sensitized *in vitro* to either Alloantigens or Tumor Antigens. *J. Immunol.*, **114**, 1083.

Kirchner, H., Chused, T. M., Herberman, R. B., Holden, H. T. & Lavrin, D. H. (1974) Evidence of Suppressor Cell Activity in Spleens of Mice Bearing Primary Tumors Induced by Moloney Sarcoma Virus. *J. exp. Med.*, **139**, 1473.

Klein, W. J., Jr (1972) Effect of Spleen Cells on Cytotoxicity by Immune Lymphnode Cells: Cell-mediated Immune Suppression (? Enhancement) *in vitro*. *J. Immunol.*, **109**, 51.

LAMON, E. W., WIGZELL, H., KLEIN, E., ANDERSSON, B. & SKURZAK, H. (1973) The Lymphocyte Response to Primary Moloney Sarcoma Virus in Balb/c Mice: Definition of Active Subpopulation at Different Times after Infection. *J. exp. Med.*, **137**, 1472.

LYNCH, R. G., GRAFF, R. J., SIRISINHA, S., SIMMS, E. S. & EISEN, H. N. (1972) Myeloma Proteins as Tumor Specific Transplantation Antigens. *Proc. natn. Acad. Sci., U.S.A.*, **69**, 1540.

MOSIER, D. E. & JOHNSON, B. M. (1975) Ontogeny of Mouse Lymphocyte Function—II. Development of the Ability to Produce Antibody is Modulated by T Lymphocytes. *J. exp. Med.*, **141**, 216.

NATHAN, C. F. & TERRY, W. D. (1975) Differential Stimulation of Murine Lymphoma Growth *in vitro* by Normal and BCG Activated Macrophages. *J. exp. Med.*, **142**, 887.

PICKAVER, A. H., RATCLIFFE, N. A., WILLIAMS, A. E. & SMITH, H. (1972) Cytotoxic Effects of Peritoneal Neutrophils on a Syngeneic Rat Tumor. *Nature, New Biol.*, **235**, 186.

POTTER, M. (1967) The Plasma Cell Tumors and Myeloma Proteins in Mice. In *Methods in Cancer Research*. Ed. H. Bush. New York and London: Academic Press.

PREHN, R. T. (1972) The Immune Response as Stimulator of Tumor Growth. *Science, N.Y.*, **176**, 170.

PREHN, R. T. & LAPPÉ, M. A. (1971) An Immunostimulation Theory of Tumor Development. *Transpl. Rev.*, **7**, 26.

ROLLINGHOFF, M. & WAGNER, H. (1973) *In vitro* Induction of Tumor Specific Immunity: Requirement for T Lymphocytes and Tumor Growth Inhibition *in vivo*. *Eur. J. Immunol.*, **3**, 471.

TAKAHASHI, T., OLD, L. J. & BOYSE, E. A. (1970) Surface Antigens of Plasma Cells. *J. exp. Med.*, **131**, 135.

UMIEL, T. & TRAININ, N. (1974) Immunological Enhancement of Tumor Growth by Syngeneic Thymus-derived Lymphocytes. *Transplantation*, **18**, 244.